# Book Reviews

**Published:** 1891-09

**Authors:** 


### Book Reviews.

DISEASES OF THE NERVOUS SYSTEM. By William A. Hammond, M. D., Surgeon-General U. S. Army (Retired List), late Professor of Diseases of the Mind and Nervous System, College of Physicians and Surgeons, New York, etc.; with the collaboration of Græme M. Hammond, M. D., Professor of Diseases of the Mind and Nervous System in the N. Y. Post Graduate Medical School, etc. Ninth Edition. D. Appleton & Co., New York.

Dr. Hammond's work on Nervous Diseases has too long been a classic to require extended review. It has been translated into several foreign languages, and is accepted as a treatise of great merit and authority wherever it is read. Some new chapters have been added in the present edition—on Acromegaly, Symmetrical Gangrene of the Extremities, etc. Several new plates and illustrations have also been inserted.

With the assistance of his son the distinguished author has brought his work fully up to date, and has disarmed adverse criticism.

---

PRACTICAL THERAPEUTICS. By Hobart A. Hare, M. D., Late Demonstrator of Therapeutics in the University of Pennsylvania; now Professor of Materia Medica and Therapeutics in Jefferson Medical College, Philadelphia, etc. Lea Brothers & Co., Philadelphia.

The author's apology for writing this work is his belief that "most of the text-books on materia medica treat of it as if the student were already a skilled physician or experimental pharmacologist." He states that his object is to furnish students with the *reasons* for the pursuance of a given policy under given conditions. This is a very laudable purpose, and throughout the book, and particularly in Part IV., we find it very well sustained. The department of Therapeutics proper is something more than a list of diseases with an annexed list of drugs which have been tried in the treatment of the same—it is an intelligent, but brief, description of the rational application of remedial agents. However, we cannot but question the application of the word *practical* to a book on therapeutics which devotes three and a half pages to Typhoid Fever, and five to Tuberculosis, and eleven pages to Epilepsy, after saying, to begin with, that it is the "most disheartening condition as to treatment that the physician has to deal with."

A few typographical errors are to be found. The one on page 338, recommending one-fifth of a grain of strychnine three times a day, might be disastrous.

